# The Precautionary Principle, Evidence-Based Medicine, and Decision Theory in Public Health Evaluation

**DOI:** 10.3389/fpubh.2016.00107

**Published:** 2016-07-07

**Authors:** Alastair J. Fischer, Gemma Ghelardi

**Affiliations:** ^1^Office for Health Economics (OHE), London, UK; ^2^London School of Economics, London, UK

**Keywords:** precautionary principle, evidence-based medicine, decision theory, effectiveness, cost-effectiveness

## Abstract

The precautionary principle (PP) has been used in the evaluation of the effectiveness and/or cost-effectiveness of interventions designed to prevent future harms in a range of activities, particularly in the area of the environment. Here, we provide details of circumstances under which the PP can be applied to the topic of harm reduction in Public Health. The definition of PP that we use says that the PP reverses the onus of proof of effectiveness between an intervention and its comparator when the intervention has been designed to reduce harm. We first describe the two frameworks used for health-care evaluation: evidence-based medicine (EBM) and decision theory (DT). EBM is usually used in *treatment* effectiveness evaluation, while either EBM or DT may be used in evaluating the effectiveness of the *prevention* of illness. For cost-effectiveness, DT is always used. The expectation in Public Health is that interventions employed to reduce harm will not actually increase harm, where “harm” in this context does not include opportunity cost. That implies that an intervention’s effectiveness can often be assumed. Attention should therefore focus on its cost-effectiveness. This view is consistent with the conclusions of DT. It is also very close to the PP notion of reversing the onus of proof, but is not consistent with EBM as normally practiced, where the onus is on showing a new practice to be superior to usual practice with a sufficiently high degree of certainty. Under our definitions, we show that where DT and the PP differ in their evaluation is in cost-effectiveness, but only for decisions that involve potential catastrophic circumstances, where the nation-state will act as if it is risk-averse. In those cases, it is likely that the state will pay more, and possibly much more, than DT would allow, in an attempt to mitigate impending disaster. That is, the rules that until now have governed all cost-effectiveness analyses are shown not to apply to catastrophic situations, where the PP applies.

## Introduction

Pregnant women and those who hope to become pregnant in the near future should take precautions so that they are not exposed to the Zika virus during or just before pregnancy. This is because of the risk of microcephaly of the fetus, even though (at the time of writing) there is no definitive scientific “proof” that the Zika virus causes the condition. That is, faced with the uncertainty of cause and effect and with a serious possibility of danger, people and communities are often advised to alter their behavior (and to spend money to do so) without the necessity of a high degree of scientific proof that would normally be required to validate an intervention.

Circumstances where precautions are taken are common. Pedestrians crossing roads, even those that are little used, still look both ways for traffic. Drivers of vehicles on public roads make defensive (i.e., precautionary) decisions on a continuous basis. No formal estimation of the statistical level of proof of danger is ever made. It is reasonable to assume that this is because the potential cost of a disastrous outcome is high, but the cost of taking precautions is generally negligible. In this paper, we are concerned with the taking of precautions to avoid dangers (at a societal level) when the costs of doing so may not be negligible. Such circumstances are the potential realm of the precautionary principle (PP).

For all that, scholars and legislators have yet to agree on a definition of the PP, which would leave no ambiguity and could thus be used as a respectable tool in public policy debates.

The 1992 Rio Declaration on Environment and Development (1), the first major official international document to recognize the PP, states that “… where there are threats of serious or irreversible damage, lack of scientific certainty shall not be used as a reason for postponing cost-effective measures to prevent environmental degradation.” In other words, in the face of reasonable concerns as to the severity of a threat, the burden of proof of whether a mitigating intervention should be carried out should not fall on those undertaking the intervention. Despite the principle having initially been associated with environmental law, it is becoming increasingly important in other areas of public policy debate. The EU incorporated it into its legislation, and it is increasingly mentioned in reference to international commerce agreements, all without a clear framework for its use having being developed (2).

The main problem with the PP is the inability to agree on the degree of precaution required and its cost, rather than the necessity of precaution *per se*. The difficulty arises because the size, probability, and timing of a potential calamity are often unknown. Supporters of a “strong” application of the principle claim that action should always be taken to prevent harm in those situations in which the threat could be severe, while those advocating a “weak” version maintain that precaution should be allowed but not required, as it should take into consideration cost-effectiveness factors and the inability of some countries to provide the necessary protection (3). The debate has been further stimulated by ethical concerns, cultural influences, and ideological differences between those believing that the application of the PP could hinder progress and those believing that preventing harm is more important for public policy (4, 5).

The PP has received very little attention from economists. It seems that at least in part, this is because it has lacked a firm theoretical basis. Quiggin (6) suggested that the PP was *necessarily* an ambiguous concept, and that a full economic analysis may not be possible due to the existence of “unknown unknowns” (7). Nevertheless, Grant and Quiggin (8) subsequently carried out a game theory analysis of the PP using a decision tree approach, where the unknown unknowns have been corralled in such a way that decisions can still allow for the PP to some extent.

This paper describes the evolution of the evaluation of health care, with particular reference to public health. It then shows how the PP can be incorporated into the decision-making process, and what difference it makes to that process. In particular, it shows that decisions based on taking the PP into account, where the intervention under consideration is designed to reduce harm, will be aligned with cost-effectiveness decisions based on decision theory (DT) (9–13) in those cases for which risk pooling is possible. However, for circumstances in which the risk is too great to be pooled, interventions to reduce harm may be approved under the PP even when the cost of precautionary action exceeds the usual cost-effectiveness thresholds.

## The Evolution of Evaluation in Health Care, with an Emphasis on Public Health

For the purposes of this paper, public health is defined as the prevention of ill health and the promotion of healthy lifestyles. A logical framework of nine criteria for defining causality and deciding (on the balance of probabilities) whether an association between variables was causative was developed by Hill (14). Since Hill wrote, there has been a huge increase in the number of randomized controlled trials (RCTs). Well-conducted RCTs can virtually eliminate biases and establish the direction of causation of the effect of an intervention. RCTs thus find themselves at the top of a hierarchy of evidence in which Hill’s other criteria play a secondary role. This is particularly true in the appraisal of health technology assessment (HTA), where RCTs are used to establish the direction of the effect of an intervention, where the estimated effect is sufficiently large (compared with its SD) to have only a specified small probability of being due to chance. This process uses only “objective” probabilities – estimated from the proportions of people involved in a particular occurrence. For example, if 160 people out of 200 in a trial respond to an experimental drug, the estimated response probability will equal 0.8. “Subjective” probabilities, such as those in the form of prior beliefs (that is, before the trial has taken place) about how effective the drug might be, will be subject to bias and will not be included as part of the objective assessment of the effectiveness of the drug. This whole process, including the hierarchy of evidence, constitutes the “external clinical evidence” that, together with the expertise of the clinician, goes under the name of evidence-based medicine (EBM) (15).

The Methods Manual for the Technology Appraisal section of the UK’s National Institute for Health and Care Excellence (16), arguably the most influential body in the world for the evaluation of health-care interventions, requires firstly that an intervention’s effectiveness be established from external evidence with sufficient certainty. In Technology Appraisal, where RCTs are routinely available, “sufficient certainty” covers the ability of RCTs to reduce biases to negligible levels and removes any consideration of prior beliefs, which would introduce bias of unknown size. In practice, other criteria generally play a role in the decision-making process only after effectiveness from RCTs has been established with sufficient certainty.

However, EBM as practiced is inconsistent with respect to the totality of outcomes. In EBM, there is generally a primary outcome, usually what is believed to be the most important of several possible types of outcome. The object of a trial is to attempt to determine whether the primary outcome when using an intervention is a sufficient improvement on the outcome without the intervention, for the improvement to be distinguished from a chance improvement. However, other outcomes (in particular, adverse events) may well be in the other direction, so that, in total, the health gain may not be positive (17). Traditional EBM does not try to add these health gains in any way. In some cases, the adverse event may be so severe as to result in death, but be rare enough for the trial to be too small to capture its true effect with any accuracy. In a truly conservative world, most interventions would thus need to be rejected, as there is an unknown possibility that they would be doing more harm than good. What tends to happen in the real world is that when rare but serious side effects occur, the positive main outcome remains isolated from other outcomes and thus is preserved. At the same time, strenuous efforts are made to enable early discovery of potential adverse events and to counter them, thus enabling the totality of the effects (if they were to be made additive) to become positive. By using this method, EBM manages to insulate itself against use of the PP.

In turn, the NICE Methods Manual for guidelines in Public Health (18) follows similar procedures with respect to external evidence, but broadens the EBM criteria to allow subjective measures of effect, essentially following the Bradford Hill criteria. In all respects, the methodology is that of DT apart from the need to ensure that an intervention has a very low probability of doing harm (see Table 1 for a comparison of EBM and DT characteristics). These measures are generally used when the appropriate RCTs are underpowered, or where RCT evidence does not exist, and include prior beliefs about the effectiveness of an intervention, the use of non-controlled studies, the role of established theory, and expert opinion about current best practice (19).

**Table 1:** **A comparison of evidence-based medicine and decision-theory characteristics for health-care evaluation**.

Evidence-based medicine	Decision theory
Only objective probabilities allowed	Subjective probabilities allowed
No prior beliefs allowed	Prior beliefs allowed
No explicit role for verified theory	Recognizes verified theory
Does not maximize aggregate health: decision maker is conservative	Maximizes aggregate health when decision maker acts as if risk-neutral
Recognizes a hierarchy of evidence with properly powered RCTs (and their meta-analysis) at the top	Recognizes the same hierarchy of evidence but is better adapted to circumstances where RCTs do not exist
Allows observation in absence of good RCT evidence	Allows observation in absence of good RCT evidence
Is ideally suited to effectiveness in health technology appraisal, particularly drug appraisal	Is better suited to effectiveness where RCTs are underpowered or cannot be undertaken, especially public health
Does not consider other projects	Takes other independent projects into account, so risk can be pooled
Used routinely in health-care research for effectiveness	Used routinely in business world for maximizing profits
Not used in health-care research for cost-effectiveness	Used in health-care research for cost-effectiveness
In practice, does not reverse onus of proof when considering harm reduction. Thus is inconsistent with its conservatism characteristic and with the precautionary principle	For harm reduction, does not reverse onus of proof but often does the equivalent *via* prior beliefs, observations, and recognition of received theory. Thus is consistent with the precautionary principle for effectiveness Is consistent with the precautionary principle for cost-effectiveness except in cases of widespread catastrophe
“Value of information” cannot be derived (not discussed in main text)	“Value of information” may be derived (not discussed in main text)

As we spell out in the next section on the PP, for interventions that are designed to reduce harm, prior beliefs, and observation (which are admissible evidence for DT) can often establish a direction of change of effectiveness with sufficient confidence, without requiring a formal demonstration *via* an RCT.

Whether the effectiveness stage of evaluation is carried out using EBM or DT, DT is always used whenever a further stage of evaluation is carried out, to establish whether an intervention that has been shown to be effective is also cost-effective. This further stage of evaluation is not always used. In the USA, evaluation of health-care interventions often stops at comparative effectiveness. In the UK, the evaluation of cost-effectiveness in health-care interventions (including those of public health) is carried out on a systematic basis by NICE. Other OECD countries tend to be somewhere between the USA and the UK in their use of this second stage.

For cost-effectiveness analysis that is able to compare disparate interventions anywhere in health care, the health effects of an intervention are converted into one of two similar generic measures known as a quality-adjusted life-year (QALY) or a disability-adjusted life-year (DALY). (The rest of the paragraph describes a QALY analysis; a DALY analysis substitutes “DALY” and “DALY lost” for “QALY” and “QALY gained,” respectively.) The additional cost of an intervention compared with usual care is divided by the health effects of the intervention (over and above those of usual care) to produce an incremental cost per QALY, known as an incremental cost-effectiveness ratio (ICER). The interventions with the lowest ICERs (if no other criteria are considered) will be the ones approved for use. The approval of interventions should stop at that level of ICER where it is thought that the health budget will have been exhausted, and that level is called the threshold ICER. In this way, the aggregate number of QALYs gained from the totality of interventions will be maximized for a given health budget. For simplicity of exposition, we shall exclude from our analysis other factors taken into account in the decision process, such as political considerations, value judgments, and social influences. These are not unimportant, but tend to be context-specific and thus are assumed to be held constant when comparing the overarching principles found in EBM, DT, and the PP.

The sampling variability of the estimated mean ICER is all but ignored, on the basis that the health-care provider will have a large number of independent projects under consideration for funding. The health-care provider is assumed to act as if it were an insurer. For example, an insurer of dwellings against fire acts on the belief that not every dwelling will be destroyed by fire at the same time. That is, they are able to pool the risk of a payout. If they have insured a very large number of dwellings, they can, in essence, ignore variability altogether: they can act as if they are risk-neutral. The assumption of risk neutrality within DT is one of its basic features (9). Thus, when applied to whether an intervention in health care should be placed on a list of interventions approved for funding, decision makers should be guided by whether the estimated mean ICER of the intervention compared with usual practice falls below the threshold ICER, with scant or zero regard to sampling variability.

The use of a generic measure of health, such as the QALY, allows health effects from a trial or observational study to be aggregated into a single index. The building blocks that allow QALYs to be estimated can be replicated across studies, which thereby reduces the subjectivity associated with estimating the overall health effects. Thus, the improvement in effects associated with the main outcome of an intervention in traditional EBM analysis can be measured by the same QALY yardstick as the health lost from adverse events. That means that DT used at the effectiveness stage of the evaluation has the potential for greater accuracy than that of EBM. However, the additional step of the tracking and collation of side effects reports, and acting on them, which is implicit in EBM analysis, is missing from DT.

The next section introduces the PP into the evaluation process.

## The Precautionary Principle: Effectiveness

The PP may be applied to many aspects of health-care evaluation. For the evaluation of most *treatments*, particularly drugs, it is not known in advance whether the treatment will give positive benefits in the form of an improved main outcome compared with a relevant comparator. EBM is conservative in that its use will not allow the use of new treatments if harm cannot be ruled out with sufficient certainty. (As mentioned in the section above, this is not always true with respect to rare side effects.) In effect, this is a PP at work. That is, there are assumed to be greater risks involved in *moving away from* the *status quo* than in staying there. The PP puts a hurdle in place to reduce the probability of harm from treatment.

Public health, in contrast, is mostly concerned with *preventing* future harm. That is, compared with treatment, where *ex ante* the intervention is feared because it may increase harm, the preventative interventions are designed to reduce harm. The conservative approach should thus be to undertake the intervention: greater risks are involved in *remaining at* the *status quo*. DT recognizes this by allowing prior knowledge about harm that may occur without intervening, assumes that the intervention reduces harm, and proceeds to determine whether the intervention is cost-effective. It is thus aligned with the PP, which makes it very difficult to favor the *status quo*. However, if EBM is used in evaluating harm-reduction interventions without reversing the onus of proof, its use is inconsistent with the conservative principles that many believe should underpin it. Thus, standard EBM, used in evaluating harm-reduction interventions, will give different answers from those of the PP and DT.

However, the preventative intervention itself may also (unwittingly or otherwise) introduce harm, so it will not always be clear whether moving away from the *status quo* is more or less conservative than remaining there. An example of the conundrum that is thereby posed is the building of new nuclear power plants. (Which of these would cause greater harm: the CO_2_ increase in staying with coal-fired generators, or the threat posed from radioactive waste and a higher probability of the proliferation of nuclear weapons?). Nevertheless, interventions, such as education or advice about, say, avoiding unsafe sex, will almost certainly reduce harm without much chance of introducing new harms. For these cases, therefore, the PP would suggest that the onus of proof should be changed: we should think of the “control” group as those people who undertake the intervention and the “treatment” group as those who receive the *status quo*.

For interventions in public health that are currently evaluated using the standard form of EBM, a change in the method of evaluation to DT or the PP could have substantial ramifications: a number of public health interventions that currently do not receive funding would do so in future. However, humans have much greater flexibility of mind than this. They change the rules when it suits them, despite the contradictions of the theory. A simple example of rule changes to suit “common sense” is given by Smith and Pell (20), who have noted that no RCT of parachutes has ever been conducted. Strict application of EBM would suggest that parachutes should not be used until an RCT has assured their safety.

This example implicitly uses DT rather than EBM. DT allows the use of prior beliefs and observation. In the above example, the prior beliefs about, or observation of, this intervention are so strong that the standard EBM rules for effectiveness can be by-passed. Threlfall et al. (21) give examples of community gardening and of volunteering for their effect on the mental health of elderly people. Trials in these areas are not straightforward: how can relatively large numbers of elderly people be successfully placed into an active gardening arm of a trial and others into a “do nothing” arm? Trials will therefore be small and thus underpowered, and almost invariably their results will be inconclusive. Yet nobody seriously believes that at a population level, the health of elderly people wishing to do gardening will be made worse off by doing so. Whether a scheme set up to place such people into gardening activities is cost-effective is entirely another matter. By suggesting the direction in which gardening would improve mental health does not necessarily mean that the scheme should be undertaken: the effect on mental health might be small and the cost of the scheme may be relatively high. That is, allocating money to improve the mental health of older people by setting up gardening opportunities might be better spent elsewhere, and that has to be estimated using cost-effectiveness analysis. When NICE produced a guideline for ways of improving the mental health of old people, however, gardening did not feature in its recommendations, because the committee considering the evidence had been informed that there was insufficient evidence to determine whether the activity would do more good than harm.

The same is true of other public health interventions involving harm reduction. For example, we can safely believe that a reduction in air pollution has a very high probability of not doing harm. (Note that here, we are talking about effectiveness and not cost-effectiveness, so we do not need to count opportunity cost as a harm.) If we apply DT rather than EBM to harm-reduction interventions, we already know the direction of change of outcomes with sufficient certainty. In practice, the DT approach of saying that in most harm-reduction circumstances, the direction of change of the effect of an intervention is known, yields the same result as undertaking an analysis where the onus of proof is reversed. That is, instead of having to show with sufficient certainty that the intervention that is designed to reduce harm is more effective than the *status quo*, the DT approach is the equivalent of having to show with sufficient certainty that the *status quo* is more effective than the intervention that is designed to reduce harm. That is, the PP does not alter conclusions reached by a DT analysis for determining *effectiveness*.

However, the PP makes a difference in an important group of cases when *cost-effectiveness* is considered. As mentioned above, we assume that the decision process is two-staged, where the determination of effectiveness is the first stage and cost-effectiveness the second, which is always conducted within decision theory.

For many circumstances within Public Health, RCTs do not and in many cases cannot exist. When this occurs and using EBM, studies such as those about interventions would be downgraded due to the hierarchy of evidence: the quality of the study would usually be called “weak.” If that criterion were the only one used, decision makers using EBM would not have been able to impose taxes on alcohol and tobacco; smoking bans in pubs, restaurants, and public transport; or make laws requiring all vehicle drivers in a country to drive on the same side of the road (19). It is clear that there is an implicit use of either DT or the PP in all such areas. All the interventions in the examples given above are designed to reduce harm.

## The Precautionary Principle: Cost-Effectiveness

We use standard text-book theory of insurance [for example, Ref. (22)] to describe what happens when the insurance function of national bodies is analyzed and show what happens when a risk cannot be insured. Sunstein (23, 24) recognizes the insurance aspects of the PP, though his analysis is less formal than ours below. We begin with individuals.

**Figure 1:**
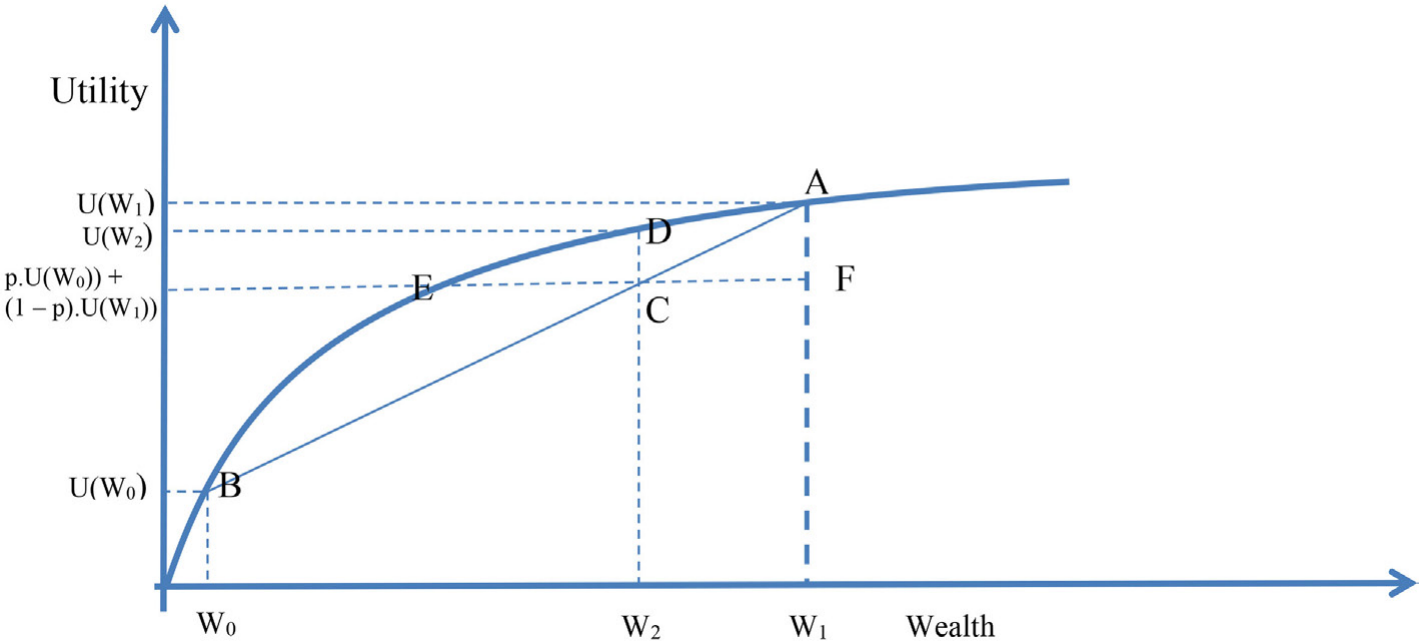
**The precautionary principle and cost-effectiveness in the absence of self-insurance**. Countries are able to self-insure against health risks. They can thereby pool risks and act as if they were risk-neutral. If a health problem becomes so great that the country is unable to self-insure, it will become risk-averse and will be prepared to pay more per unit of health gain than if it is able to self-insure.

Figure 1 describes the relationship between wealth and the marginal utility of wealth. That is to say: a rich man will value the last dollar that he has earnt less than the last dollar earnt if he were poor. That is, the marginal utility of a person’s last dollar becomes smaller as the number of dollars that the person owns increases.

Suppose a person’s wealth is given as *W*_1_, the person’s utility of wealth is thus *U*(*W*_1_). We denote the position [*W*_1_, *U*(*W*_1_)] in Figure 1 by A. Suppose that most of the person’s wealth is in the form of a home, and that the remaining wealth is given by *W*_0_. Its utility is *U*(*W*_0_); and [*W*_0_, *U*(*W*_0_)] is given by B in Figure 1. Suppose that the probability of the home being destroyed is *p*, so the probability of its not being destroyed is (1 − *p*). Beforehand, not knowing whether the home will be standing or destroyed, the person’s average utility is the average of *U*(*W*_0_) and *U*(*W*_1_) weighted by the probabilities *p* and (1 − *p*), and is thus *p* × *U*(*W*_0_) + (1 − *p*) × *U*(*W*_1_). We call the corresponding level of wealth *W*_2_. We denote the point in Figure 1 as C. This is the utility of the uninsured level of wealth. If the person were to pay *W*_1_ − *W*_2_ (=CF in Figure 1) as a fair insurance policy beforehand, and the home were subsequently destroyed, the person would have a utility of wealth *U*(*W*_2_), given as D in Figure 1. This is because the insurance policy allows the home to be rebuilt. The distance DC is the increased utility of having the insurance. The distance EC is known as the risk premium. EF is the largest premium that a person would pay for insuring their home, because at E, the person who insures has the same after-the-event utility as the person who is uninsured and whose home is destroyed. Thus, the person is indifferent between insuring and not insuring when they have to pay EF as a premium.

We now apply the analysis of the PP to this situation. In so doing and without any loss of generalization, we change what we said with respect to an individual in the above paragraph to a national body such as a national health service.

The national body self-insures where it can. If instead of being home insurance, now suppose that Figure 1 applies to a Public Health scenario. In “normal” circumstances, the nation’s wealth would be given by *W*_1_. In catastrophic circumstances, it would be *W*_0_. A self-insuring national body would be prepared to pay an amount CF for an intervention to avoid *W*_0_. When it is able to self-insure, CF represents the most that the national body needs to pay for the intervention and thus represents the intervention’s threshold cost. However, some things would be too big to insure against. A nation would not be able to go to other nations to insure against a large asteroid strike on the earth, or protect against global warming or antibiotic resistance, because all nations would have the same or similar risk. However, in terms of orders of magnitude, we can discern from Figure 1 what a nation might be prepared to pay when it can no longer be risk-neutral. By being unable to self-insure, the nation has become risk-averse. In Figure 1, spending as little as CF to receive full insurance cover is no longer possible. The nation unable to self-insure would pay up to EF. As drawn, EF is more than double CF, but for absolute catastrophes that might spell the end of the species without an intervention, the concavity of the utility of wealth function would increase. If so, a nation would presumably be willing to fund projects whose cost to benefit ratio were to be greatly in excess of the ratio EF:CF as shown in the Figure.

It can thus be argued that the PP for events that a national health-care insurer can self-insure against will make no difference to the threshold ICER. When a nation is unable to self-insure, however, the usual rules of cost-effectiveness, which presuppose a national body that acts as if it is risk-neutral, do not apply. The amounts that nations will be prepared to pay to avoid global warming or antimicrobial resistance may well greatly exceed “normal” cost-effectiveness thresholds.

For relatively minor calamities, nations may not be able to self-insure but may be able to buy insurance from other nations. It may be possible to gain some idea of the risk premium that might be needed to be paid by looking at nations that may default on international debts. The higher the probability of default, the greater the interest rate payable on the debt.

## Conclusion

This paper examines what happens when the PP is applied to health-care interventions.

If the first stage of a decision-making process (the determination of effectiveness) is to be conservative, then EBM is inconsistently applied if it fails to recognize that harm reduction is usually a conservative response.

The paper shows that there exists a vast array of circumstances where the PP may potentially be used, but that it does not change many decisions of national health-care insurers if they are already using a decision-theory approach for both effectiveness and cost-effectiveness. However, if they are using EBM at the effectiveness stage in an area of harm reduction, there may be insufficient evidence to determine whether an intervention is effective. In this case, the application of the PP will often result in different decisions being made. In other words, the PP is consistent with a decision-theory approach to effectiveness when looking at harm reduction, but not consistent with an EBM approach.

Within a decision-theory framework, most health-care insurers are able to act as if they are risk-neutral by pooling risk, as all insurers do. When self-insurance is possible, the PP and cost-effectiveness analysis reach the same conclusion about whether an intervention is worthwhile. However, when a risk becomes so great that an insurer at the national level is no longer able to self-insure, the amounts spent on an intervention depend on the extent of risk aversion that the nation displays. The decision-theory rules of cost-effectiveness break down in such circumstances, as they assume risk neutrality. For that reason, it would not be possible to determine whether interventions to mitigate climate change or antimicrobial resistance are cost-effective: the nation itself must decide independently of economists the amounts it is prepared to spend to avoid catastrophe. Economists can offer useful advice only if they are able to measure a nation’s risk aversion under dire circumstances. The PP recognizes this but requires an estimate of the extent of a nation’s risk aversion to enable an estimate to be made of the size of the amounts that should be spent to avoid large-order catastrophes. Since the level of risk aversion will depend on the size of the catastrophe, it is unlikely that reliable estimates can be made of the amounts that should be spent, given that the catastrophe has not yet occurred. What this paper does is to alert authorities that it would not be appropriate to try to limit expenditure to threshold-cost-per-QALY amounts to counter catastrophes.

There may be a middle ground where absolute calamity is not likely, but where self-insuring is difficult. An example of this could be an epidemic caused by an infectious disease that overloads medical facilities and thus displaces health care that would normally be available. If a country in this situation had to borrow overseas to pay for the costs of treating the epidemic, it may have to pay a premium on the repayments, to cover the risk of default. This would relate to the extent to which breaking the cost per QALY threshold would be required.

Perhaps rather more importantly, the paper is able to answer the questions of when to act, given that by reversing the onus of proof, the PP will now establish the effectiveness of some interventions that could not previously be established. However, cost-effectiveness must then also be established before a positive decision to act can be made. Reversing the onus of proof is in essence what DT also does in the same circumstances in public health appraisal, so both PP and DT are very likely to establish effectiveness in more cases than using standard EBM. However, the argument about PP not being affordable if it is used everywhere is a spurious one, because it will only be used where it is cost-effective to do so.

The paper also is able to sort out the arguments about weak and strong versions of the PP. The weak version says that cost-effectiveness should be taken into account when deciding whether to act using the PP as the effectiveness criterion, and funding should go only to those interventions that satisfy cost-effectiveness. Our analysis reaches the same conclusion. However, when a threat or its expectation is sufficiently severe, the strong version says that action should be taken regardless of cost-effectiveness. Our analysis also gives qualified support for this proposition, by loosening the rules for cost-effectiveness: the greater the threat, the greater a nation’s risk aversion, so the looser the cost-effectiveness threshold becomes. Moreover, we indicate in terms of insurability against risk where the weak rule stops and the strong rule begins to take effect.

This paper also bolsters the Bradford Hill’s view of how decisions about public health interventions should be evaluated, by placing his criteria into a decision theoretic framework. It then shows that this framework is in accord with the PP in most circumstances but would need to be modified in the face of calamities.

## Author Contributions

AF devised the topic, developed the theory, and wrote much of the paper. GG wrote the Introduction based on a literature review of the topic which she undertook and helped in revising the paper. The work for this paper was carried out during AF’s previous employment in the Public Health Section of the National Institute for Health and Care Excellence (NICE), where GG was a student on secondment from LSE.

## Conflict of Interest Statement

The authors declare that the research was conducted in the absence of any commercial or financial relationships that could be construed as a potential conflict of interest.
